# Fracture events in patients with primary biliary cholangitis during treatment with seladelpar in the phase III RESPONSE trial

**DOI:** 10.1097/HC9.0000000000000845

**Published:** 2025-11-20

**Authors:** John M. Vierling, Gideon M. Hirschfield, Robert P. Kustra, Xiangyu Liu, Sarah Proehl, Daria B. Crittenden

**Affiliations:** 1 Departments of Medicine and Surgery, Baylor College of Medicine, Houston, Texas, USA; 2 Department of Medicine, The Autoimmune and Rare Liver Disease Programme, Toronto General Hospital, Toronto, Ontario, Canada; 3 Gilead Sciences, Inc., Foster City, California, USA

**Keywords:** bone diseases, metabolic, liver cirrhosis, biliary, osteoporosis, peroxisome proliferator–activated receptor delta, vitamin D deficiency

## INTRODUCTION

Primary biliary cholangitis (PBC) is a chronic, cholestatic, autoimmune disease associated with progressive liver injury that predominantly impacts women.^1^ Osteoporosis, a disease of low bone density and increased risk for fracture, is most commonly observed in postmenopausal women and men over the age of 50.^2^ Osteoporosis is often underdiagnosed and undertreated, even after an initial fracture, which increases the risk for subsequent fractures.^2^ Chronic cholestasis, regardless of etiology, is an independent risk factor for osteopenia and osteoporosis.^1^,^2^ Accordingly, fracture rates are higher among patients with PBC compared with age-matched and sex-matched controls.^3^ PBC management guidelines recommend baseline bone mineral densitometry and serum calcium and vitamin D levels, with annual retesting of serum vitamin D levels and a repeat bone mineral densitometry every 2 years.^1^

Seladelpar, a first-in-class delpar (ie, a selective and potent peroxisome proliferator–activated receptor delta [PPARδ] agonist), was approved in 2024 as second-line therapy for PBC.^4^ In the pivotal phase III, placebo-controlled RESPONSE trial (NCT04620733), 193 patients with PBC and an inadequate response or intolerance to ursodeoxycholic acid were randomized 2:1 to receive once-daily seladelpar 10 mg or placebo for 12 months.^4^ Seladelpar was overall safe and well-tolerated and led to significant reductions in biochemical markers of cholestasis, as well as to statistically significant and clinically meaningful improvements in pruritus.^4^

During RESPONSE, 5 patients (4%) receiving seladelpar experienced a fracture, while no fractures occurred in patients receiving placebo. This report summarizes baseline characteristics of bone health among all patients enrolled in RESPONSE and provides detailed assessments of the 5 cases of fracture observed during the study.

## RESPONSE STUDY METHODS

Details of the study design have been previously published.^4^ Written consent was obtained from all patients. This study was approved by regulatory authorities and institutional review boards or ethics committees at each site and was conducted in strict accordance with the Declaration of Helsinki and International Conference on Harmonization Good Clinical Practice guidelines. Medical history and prior medication use were collected at baseline and included information on osteopenia, osteoporosis, vitamin D deficiency, and prior osteoporosis treatments. Fractures were collected as adverse events.

### Population bone health at baseline

In RESPONSE, baseline PBC disease severity was similar between patients randomized to seladelpar (n=128) or placebo (n=65), as reported.^4^ Average age (57 y) and proportion of patients of female sex (~95%) were similar between the seladelpar and placebo groups. Mean 25-OH vitamin D levels were normal at baseline in both the seladelpar (40.9 ng/mL) and placebo (36.2 ng/mL) groups (Table 1). However, the seladelpar group had numerically more patients with a prior history of osteoporosis, vitamin D deficiency, and osteoporosis treatment than did the placebo group (Table 1).

**TABLE 1:** Baseline bone health characteristics by treatment group in the RESPONSE trial

	Seladelpar 10 mg, n=128	Placebo, n=65
Osteopenia^a^	17 (13)	8 (12)
Osteoporosis^b^	18 (14)	5 (8)
Vitamin D deficiency^c^	23 (18)	3 (5)
25-OH vitamin D, ng/mL, mean	40.9	36.2
Past medical history of fracture^d^	7 (5)	4 (6)
Receiving treatment for osteoporosis^e^	8 (6)	2 (3)

*Note*: Data presented as n (%), unless otherwise stated.

^a^ Search strategy included the preferred term of osteopenia.

^b^ Search strategy included the preferred term of osteoporosis.

^c^ Search strategy included the preferred term of vitamin D deficiency.

^d^ The fracture FMQ search strategy (with removal of the preferred term tooth fracture) was used.

^e^ Search strategy included any concomitant medications for the indication of osteoporosis.

Abbreviations: FDA, Food and Drug Administration; FMQ, FDA Medical Query.

### Case details of patients with fractures and exposure-adjusted fracture incidence

Clinical details for the 5 patients (4%) who experienced fractures while taking seladelpar during RESPONSE are summarized in Table 2. Fractures were classified as likely osteoporotic if they occurred in the setting of a fall from a standing height or less (or equivalent) and at anatomical sites where low bone mineral density increases fracture risk, based on UK NICE guidelines. This excludes fractures of the fingers, toes, and facial bones.^5^ In the absence of information on the degree of trauma, fractures at relevant anatomical sites were designated as possibly osteoporotic in nature. The exposure-adjusted observed fracture incidence in the seladelpar arm of RESPONSE was 4.1 fractures per 100 patient-years.

**TABLE 2:** Demographic and baseline clinical characteristics, past medical history, and fracture details of patients who reported fractures during the RESPONSE trial

Case	Cirrhosis status^a^ and liver stiffness (kPa)^b^	Age	Sex	Postmenopausal?	General medical history	Relevant bone history	Relevant concomitant medications	Relevant laboratory assessments^c^	Fracture site^d^	Fracture details and study day of the event	Osteoporotic fracture?
1	Noncirrhotic, 16.1	55	F	Y	Vitamin D deficiency, vertigo, and sleepwalking	History of left distal radius fracture 6 months before study start	Omeprazole, alprazolam, venlafaxine, cholecalciferol, topiramate, propranolol, and lisinopril	ALP: 299 TB: 0.62 eGFR: 74.72 25-OH-D: 58	Humerus	No mechanism details, day 164	Possible
2	Cirrhotic, 27.0	74	F	Y	Hyperthyroidism and atrial fibrillation	Osteoporosis and a history of bilateral hip replacements	Thiamazole, bisoprolol, dabigatran, metoprolol, atorvastatin, flecainide, and ramipril	ALP: 506 TB: 1.21 eGFR: 105.56 25-OH-D: 36	Femur	Femur fracture after a fall from standing height in the backyard, day 349	Y
3	Cirrhotic, 16.5	72	F	Y	NA	Osteopenia	Omeprazole, calcium carbonate, cholecalciferol, zolpidem, cyclobenzaprine, amlodipine, spironolactone, escitalopram, and atorvastatin	ALP: 322 TB: 0.61 eGFR: 97.14 25-OH-D: 67	Heel	No mechanism details, day 295	Possible
4	Noncirrhotic, 9.4	44	F	N	NA	Osteopenia	Ferrous glycine sulfate and calcifediol	ALP: 267 TB: 0.48 eGFR: 112.25 25-OH-D: 42	Toe	No mechanism details, day 89	N
5	Noncirrhotic, 10.4	69	M	NA	Stage 3 chronic kidney disease	NA	Hydrocodone, famotidine, rabeprazole, bupropion, atorvastatin, celecoxib, diazepam, vitamin D, cyclobenzaprine, and hydroxyzine	ALP: 198 TB: 0.78 eGFR: 44.20 25-OH-D: 39	Wrist	Broken wrist due to a motor vehicle accident, day 296	N

^a^ Cirrhosis was defined per-protocol and included: liver biopsy demonstrating cirrhosis; clinical sequelae of cirrhosis; liver stiffness >16.9 kPa by FibroScan; combination of platelets <140×10^3^/µL with serum albumin <3.5 g/dL, INR >1.3 (in the absence of antithrombotic agent), or total bilirubin >1.0× ULN; radiological evidence of cirrhosis with concurrent splenomegaly; or clinical determination by the investigator.

^b^ Liver stiffness measurement was performed using transient elastography via FibroScan or a similar machine.

^c^ ALP reported in U/L; ULN=116 U/L. TB reported in mg/dL; ULN=1.1 mg/dL. eGFR reported in mL/min/1.73 m^2^; LLN <90 mL/min/1.73 m^2^. 25-OH-D reported in ng/mL; LLN=30 ng/mL.

^d^ As fractures were reported by patients, the specific anatomic location was not always available.

Abbreviations: 25-0H-D; 25-hydroxyvitamin D; eGFR, estimated glomerular filtration rate; F, female; INR, international normalized ratio; LLN, lower limit of normal; M, male; N, no; NA, not applicable; TB, total bilirubin; ULN, upper limit of normal; Y, yes.

## DISCUSSION

Of the 5 fractures observed with seladelpar use in RESPONSE, 3 were considered to be likely or possibly osteoporotic, while 2 were assessed as nonosteoporotic. The 3 patients with fractures assessed as likely or possibly osteoporotic had notable risk factors for fracture, including either a history of osteopenia, osteoporosis, or a recent fracture. Two of these patients also had baseline cirrhosis, which puts them at increased risk for metabolic bone disease, falls, and injuries.^6^ The fracture events in this trial showed no pattern with respect to anatomic location or duration of seladelpar treatment and occurred in the setting of a higher baseline incidence of osteoporosis in the seladelpar group versus the placebo group.

Imbalance in fractures has not been observed in other placebo-controlled seladelpar studies. In the phase III, placebo-controlled ENHANCE study that assessed seladelpar doses of 5 and 10 mg in patients with PBC, the rate of fractures was similar in the seladelpar (n=3 [2%]) and placebo (n=1 [2%]) groups, although the duration of follow-up was on average only 17.7 weeks.^7^ In a placebo-controlled study of seladelpar in patients with NASH, the incidence of fractures was similar across treatment groups during a mean follow-up of 48 weeks: 10 mg, n=1 (2%); 20 mg, n=1 (2%); 50 mg, n=3 (6%); placebo, n=2 (7%).^8^

Regarding the mechanism of action for seladelpar, agonism of PPARδ has been shown to reduce bile acid synthesis, beneficially affect lipid synthesis and metabolism, and have anti-inflammatory effects on macrophages, including KCs.^9^,^10^ Based on nonclinical data, PPARδ agonism has not been considered to promote fracture risk. Studies in mice have suggested that PPARδ agonism could potentially enhance bone formation by affecting osteoblast metabolism, although the relevance to humans is unknown.^11^

In the United States, the prevalence of osteopenia among women aged ≥50 years is 52%, and the prevalence of osteoporosis of the femoral neck or lumbar spine is estimated to be 20%.^12^ The annual incidence of fractures among predominantly White women in this age group in the United States is 3% per year.^13^ In a recent cohort study of 3980 Swedish patients with PBC (median age, 64 y), the annual fracture incidence was 5%, almost twice that of age-matched controls.^3^ Thus, the exposure-adjusted observed fracture incidence in RESPONSE (4.1 fractures per 100 patient-years) is consistent with the expected incidence for this population. In further support that the fracture rate observed was consistent with epidemiologic rates, the incidence of fractures was stable or decreased over time with continued seladelpar exposure. Patients who completed RESPONSE could enroll in the open-label ASSURE study (NCT03301506),^14^ and those continuing seladelpar in ASSURE had an exposure-adjusted patient incidence of fractures of 3.0 and 2.7 fractures per 100 patient-years in years 2 and 3 of exposure during the open-label extension study, respectively (Gilead Sciences, Inc., unpublished data, August 2025).

## CONCLUSIONS

In the RESPONSE trial, there was an imbalance in fracture events in patients receiving seladelpar versus placebo. The fracture incidence was similar to the expected annual incidence among patients with PBC of a similar age. All fractures considered likely or possibly osteoporotic occurred in patients with a history of osteopenia, osteoporosis, or prior fracture. Scrutiny of the available data did not detect a causal relationship between seladelpar and fractures. Clinicians should follow PBC management guidelines for bone health assessment and management of vitamin D deficiency and osteoporosis in patients with PBC.^1^
